# Inclusion of Language Preference in Clinical One-Liners and its Association with Use and Documentation of Interpreter Services Among Inpatients with non-English Language Preference

**DOI:** 10.1186/s12913-026-14596-x

**Published:** 2026-04-21

**Authors:** Miguel A. Linares, John Novoa-Laurentiev, Alexander Chaitoff, Nuoya Jiang, Sigfredo Salguero, Yilu Ma, Li Zhou

**Affiliations:** 1 Division of General Internal Medicine and Health Services Research, University of California, Los Angeles, CA USA; 2 Division of General Internal Medicine, Brigham and Women’s Hospital, Boston, MA USA; 3 Department of Internal Medicine, University of Michigan Medical School, Ann Arbor, MI USA; 4 Harvard Medical School, Boston, MA USA

**Keywords:** One-liner, NELP, Non-English language preference, Interpreter services, Language access, Clinical documentation

## Abstract

**Background:**

Patients with non-English language preference (NELP) experience disparities in care quality and outcomes, often due to underuse of professional interpreter services. One proposed strategy to address this gap is incorporating patients’ language preference into prominent sections of clinical documentation, such as the one-liner (OL) of clinical notes. Whether this approach is associated with increased subsequent use and documentation of professional interpreter services has not been empirically evaluated.

**Methods:**

We conducted a retrospective cohort study of 2,336 general medicine admissions for 1,487 adult patients with self-reported NELP at a large academic medical center between January 1, 2019, and December 31, 2023. We used natural language processing and large language model tools to identify the presence language preference in the OL of admitting H&P notes and to extract documentation of interpreter services use from clinical notes. The primary outcome was the rate of interpreter service encounters per admission-day. Secondary outcomes included note-based documentation of interpreter services use. Multilevel binomial and logistic regression models adjusted for demographics and clinical characteristics.

**Results:**

Language preference was documented in the OL of H&P notes in 21.2% of admissions. In adjusted analyses, inclusion of language preference in the OL was associated with 16% higher rate of interpreter service encounters per admission-day (adjusted incidence rate ratio [AIRR] 1.16, 95% CI 1.03–1.31) and increased odds of interpreter services use documentation across all notes (adjusted odds ratio [AOR], 1.71; 95% CI 1.40–2.08). Effect modification by language was observed for documentation outcomes, with higher odds observed among non-Spanish-speaking patients (AOR, 2.96; 95% CI, 2.18–4.01).

**Conclusions:**

Inclusion of a patient’s language preference in the OL of an admitting note was associated with higher interpreter encounter rates and improved documentation during hospitalization. Incorporating language preference into core clinical documentation, alongside existing EHR flags, may represent a scalable, low-burden approach associated with increased interpreter engagement. Prospective studies are needed to evaluate whether this practice improves patient-centered outcomes and can be implemented consistently across care settings.

## Background

Approximately 26 million individuals in the U.S. have non-English language preference (NELP), a generally understudied social determinant of health (SDOH) [1] associated with suboptimal outcomes across multiple healthcare domains, including safety, quality and effectiveness of care [2–4]. To bridge communication barriers stemming from language discordance, professional interpreters are essential [5, 6]. However, evidence indicates that their use is often limited and inconsistent [7].

Numerous factors contribute to variability in use of professional interpreter services (henceforth interpreter services). These include challenges faced by individual providers, such as time pressures resulting in attempts to “get by” without an interpreter [8–10], and institutional limitations, such as poorly disseminated policies and insufficient investment in interpreter services. In response, prior work has emphasized strengthening both internal (ie, the healthcare organization) and external structures (ie, factors beyond it, such as policy makers or healthcare payers) [11]. Increasing clinician awareness of patients’ language preference prior to encounters represents one strategy to strengthen internal structures of language access. For example, some studies have used alerts in the electronic health record (EHR) to make language preference more salient [12, 13]. However, with alert fatigue well-documented, alternative strategies may be needed to increase language preference awareness [14].

The one-liner (OL) in a medical note is a concise summary highlighting key information relevant to a patient’s care. Although there is no standardized set of required elements, OLs commonly include sociodemographic information, past medical history, and the presenting condition. Present in most medical notes, the OL serves as a critical reference point for clinicians, supporting information synthesis and effective interprofessional communication. Unlike EHR alerts, adding a patient’s preferred language to the OL (eg, “78-year-old Vietnamese-speaking female presenting with chest pain”) represents a non-interruptive, low-burden cue that is readily visible during chart review and bedside care. By increasing the visibility of language needs, this practice could prompt earlier and more consistent use of interpreter services. Despite this potential and prior proposals to improve language access via documentation strategies, the relationship between language inclusion in the OL and interpreter utilization has not been empirically examined [15].

In this retrospective cohort study, we assessed whether inclusion of language preference in OLs was associated with subsequent use and documentation of interpreter services.

## Methods

### Data, study population, and design

**Fig. 1:**
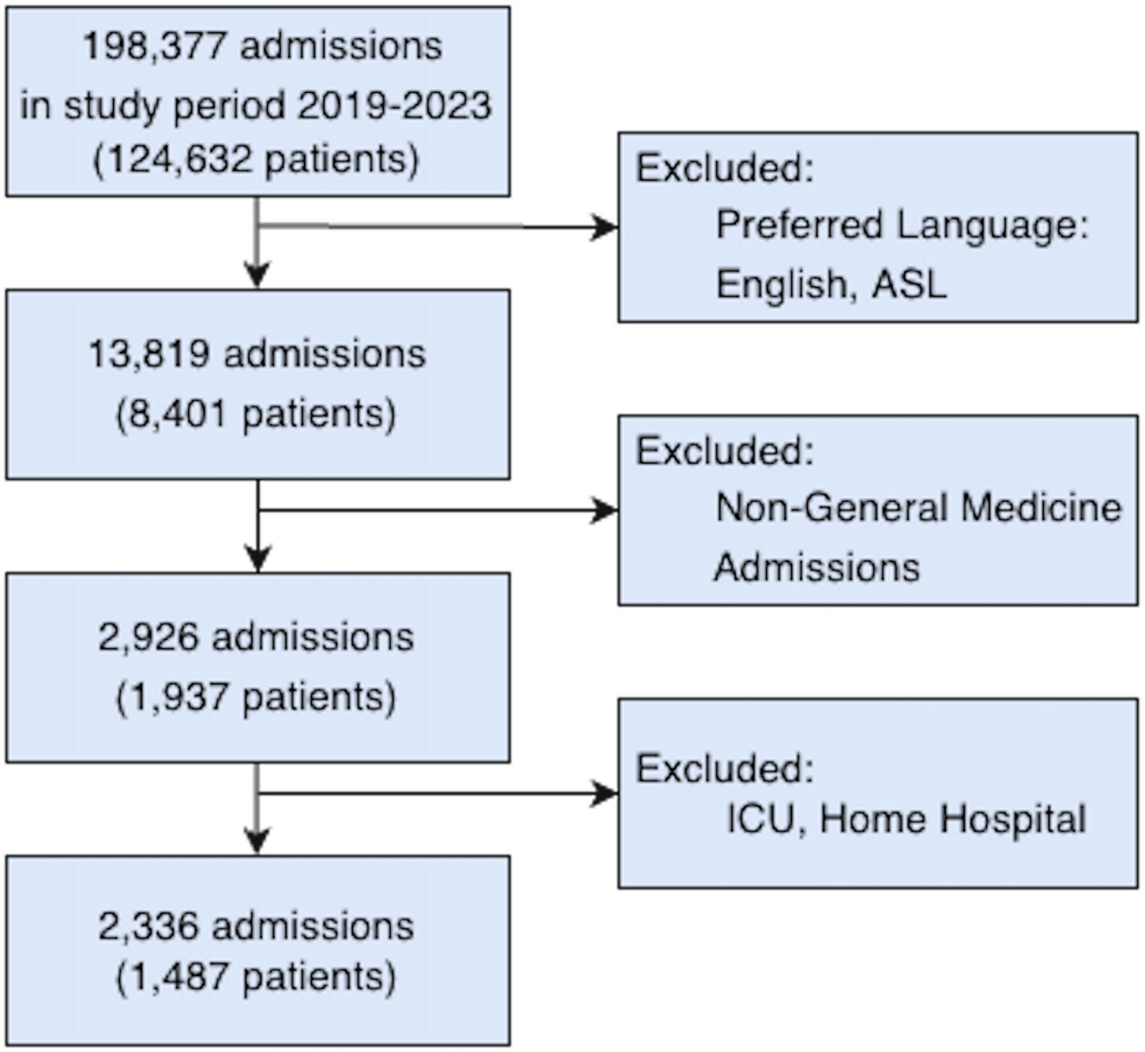
Study cohort selection flow diagram

The investigation was conducted at a single academic medical center within the Mass General Brigham (MGB) healthcare system in Boston, Massachusetts. Using the MGB Enterprise Data Warehouse (EDW) that stores Epic EHR data [16], we identified adult patients (≥ 18 years) with self-reported NELP who were admitted to the general medicine service between January 1, 2019 and December 31, 2023 (Fig. 1). Patients self-report their preferred language during the registration process and this information can be updated at any point during care. Patients admitted to the Intensive Care Unit (ICU) or Home-Hospital program were excluded. We limited the dataset to notes authored by medical students, residents, nurse practitioners (NPs), physician assistants (PAs), or attending physicians. For eligible admissions, we obtained medical notes associated with the hospital admissions from the MGB Research Patient Data Registry (RPDR), which integrates patient demographics, clinical encounters, medication history, procedures, and billing information. This study was approved by the MGB Institutional Review Board.

### Exposure

The primary exposure was clinician-level documentation of a patient’s language preference in the OL of the general medicine admitting team’s first note (ie, the history and physical note, henceforth “H&P”). We restricted the exposure to the admitting H&P OL to ensure the exposure preceded subsequent interpreter encounters; additionally, OLs in progress notes are often copy-forwarded, making them less suitable for defining a consistent unbiased exposure. OLs were identified using a rule-based natural language processing (NLP) approach [17] with regular expressions to extract them from medical notes throughout the admission (precision: 99.5%, recall: 98% on evaluating 800 randomly selected cases). Subsequently, using a structured prompt, we used GPT-4, a generative pre-trained transformer large language model (LLM, deployed within the MGB HIPAA-compliant Microsoft Azure environment) to classify whether language preference appeared in each OL (precision: 98%, recall: 100% on evaluating 1,000 randomly selected cases).

Separately, all patients with NELP had an EHR language flag indicating preferred language. This system-level indicator was present regardless of clinician documentation and applied uniformly throughout the study period. The exposure of interest was clinician inclusion of language preference within the H&P OL, independent of the existing EHR language flag.

### Outcomes

All patients with NELP admitted to this academic medical center have access to interpreter services, including in-person interpretation for a selected number of languages and remote (audio or video) interpreters through a contracted vendor for most spoken non-English languages (NEL) in the US.

#### Primary outcome

The primary outcome involved the total number of interpreter service encounters per admission as captured through the hospital’s interpreter services billing records. These records include all modalities of interpretation. Although interpreter services are federally mandated, their costs are institutionally funded and are not usually reimbursed by Centers for Medicare & Medicaid Services or private insurance payers; importantly, these services are not billed to patients. We matched unique patient and/or admission identifiers with billing dates to quantify the total number of interpreter service encounters used during each admission. We excluded 2019 data from analysis due to differences in internal billing structure for remote services during that year.

#### Secondary outcomes

Secondary outcomes included the documentation of interpreter service use within clinical notes. We operationalized this by identifying references to interpreter services using the roots “interpret” or “translat” in clinical notes and extracting surrounding context. GPT-4 was then prompted to classify whether interpreter services were documented (present or absent) based on the extracted text (precision and recall were 100% for both documentation of interpreter services use and documentation indicating absence of interpreter services). Unlike OLs, copy-forwarding of interpreter services use documentation was less prevalent as it was extracted from the highly variable “subjective” portion of the note in virtually all cases.

### Covariates

For each note, we extracted the author and associated medical team and categorized it as an H&P or progress note. Patient-level covariates included age, gender, preferred language, year of service, insurance type (public vs. private), education level, marital status, and race and ethnicity. Given that illness severity can influence the number of interpreter encounters during admission, we also included length of stay (LOS), the Charlson Comorbidity Index (CCI), number of RN notes per day, and number of non-medicine specialty teams involved in the admission [18]. We also included an indicator variable for a pre-/post–August 2020 EHR interface change, during which the primary display of sociodemographic and clinical information, including language preference, was reorganized from a horizontal header to a vertical sidebar within the chart interface.

### Statistical analysis

Descriptive statistics summarized baseline characteristics and interpreter encounters per admission, stratified by whether language preference was included in the H&P OL.

#### Primary outcome: interpreter service encounter rates

We used multilevel negative binomial regression to estimate the association between inclusion of language preference in the H&P OL and the rate of interpreter service encounters during admission. Since the outcome was a count variable and varied by admission duration, LOS (admission days) was included as an exposure term (offset) to model interpreter service encounters per admission-day. Both the primary and secondary models adjusted for a shared set of patient and admission characteristics: age, gender, language category (Spanish vs. non-Spanish), education level, marital status, year of service, insurance type, CCI, pre-/post-EHR interface change, number of non-medicine teams involved in admission, as well as average number of RN notes per day [18]. A unique patient identifier was included as a level-2 random effect to account for correlation of multiple admissions within individuals. To assess effect modification by language, we included an interaction term between inclusion of language preference in the H&P OL and language category (Spanish vs. non-Spanish. Results are presented as incidence rate ratios (IRRs) with corresponding 95% confidence intervals. Variance inflation factor (VIF) analysis was performed to assess multicollinearity among covariates, with values > 5 indicating high collinearity. Additionally, correlation coefficients were calculated, with values > 0.7 considered indicative of collinearity. Race and ethnicity were removed from the model due to collinearity with language.

#### Secondary outcomes: documentation of interpreter services

For documentation outcomes, we used multilevel logistic regression to estimate associations between inclusion of language preference in the H&P OL and documented use of interpreter services across all notes. We also conducted subgroup analysis for notes authored by specialty services. In addition to the shared covariates described above, the secondary model also adjusted for note type (H&P vs. progress notes), and medical specialty category. Random effects included author identifiers to adjust for intra-physician correlation, admission identifiers to account for similarities due to the same admission, and unique patient identifiers for correlation of admissions within individual patients. We also assessed effect modification by language by including an interaction term between inclusion of language preference in the H&P OL and language category (Spanish vs. non-Spanish). Results are reported as odds ratios (ORs) with 95% CIs.

All statistical tests were two-sided, with significance set at α = 0.05. Analyses were conducted using Stata version 17.0. We followed the Strengthening the Reporting of Observational Studies in Epidemiology (STROBE) reporting guideline for cohort studies.

## Results

### Patient-level characteristics

The study included 1,487 patients with NELP who had 2,336 inpatient admissions between 2019 and 2023 (Table 1). Most patients were female (57.6%) and had public insurance (61.2%). The mean age was 70.5 years (SD 16.7). The cohort included 123 (8.3%) Asian, 142 (9.6%) non-Hispanic Black, 906 (60.9%) Hispanic, 242 (16.3%) non-Hispanic White, and 74 (5.0%) patients categorized as Other. The medical record captured 47 unique preferred languages, the five most common were Spanish (61.5%), Russian (8.2%), Haitian Creole (5.9%), Chinese (5.1%), and Arabic (3.8%).

**Table 1:** Baseline patient characteristics by inclusion of language preference in the H&P one-liner

Characteristic	Patients, No (%)			
	All *n* = 1,487	Language Preference absent in the H&*P* One-Liner *n* = 1,157	Language Preference present in the H&*P* One-Liner *n* = 330	*p*-value (t/χ²)
Age, SD	70.5 (16.7)	69.8 (16.8)	72.8 (16.0)	< 0.01
Sex				0.92
Female	857 (57.6%)	666 (57.6%)	191 (57.9%)	
Male	630 (42.4%)	491 (42.4%)	139 (42.1%)	
**Race and Ethnicity**				0.15
Non-Hispanic Asian	123 (8.3%)	94 (8.1%)	29 (8.8%)	
Non-Hispanic Black	142 (9.5%)	108 (9.3%)	34 (10.4%)	
Hispanic	906 (60.9%)	724 (62.6%)	182 (55.4%)	
Non-Hispanic White	242 (16.3%)	177 (15.3%)	65 (19.7%)	
Other	74 (5.0%)	54 (4.7%)	20 (6.1%)	
**Insurance**				
Commercial	577 (38.8%)	457 (39.5%)	120 (36.4%)	0.3
Public	910 (61.2%)	700 (60.5%)	210 (63.6%)	
**Marital Status**				0.05
Married	587 (39.5%)	462 (39.9%)	125 (37.9%)	
Single	430 (28.9%)	346 (30.0%)	84 (25.5%)	
Separated or Divorced	202 (13.6%)	150 (13.0%)	52 (15.8%)	
Widowed	251 (16.9%)	183 (15.8%)	68 (20.6%)	
Other †	17 (1.1%)	16 (1.4%)	1 (0.3%)	
**Education**				0.58
College or Higher	184 (12.4%)	139 (12.0%)	45 (13.6%)	
Some College	60 (4.0%)	44 (3.80%)	16 (4.8%)	
High School Equivalent	436 (29.3%)	350 (30.3%)	86 (26.1%)	
Less than High School	713 (48.0%)	551 (47.6%)	162 (49.1%)	
Other †	94 (6.3%)	73 (6.3%)	21 (6.4%)	
**LOS (days)**				
	8.5 (10.2)	8.9 (10.9)	7.4 (7.7)	0.02
**CCI mean**,** SD**	1.6 (2.4)	1.6 (2.3)	1.7 (2.6)	0.66

† Includes cases when information was unavailable or the patient declined to provide this information

### Note-level characteristics

A total of 24,518 notes were analyzed, of which 23,054 (94.0%) contained an identifiable OL. These notes were authored by 1,985 clinicians; 58% of admissions included at least one note by 41 unique specialist teams. Of all notes with an identifiable OL, 10,449 (45.3%) were written by residents, 5,373 (23.3%) by PAs, 2,971 (12.9%) by attendings, 3,029 (13.1%) by clinical fellows, 1,101 (4.8%) by medical students, and 131 (0.6%) by NPs. The OL documented language preference in 21.2% (4,882) of cases. Most notes (16,441, 71.3%) were written by medicine teams, the four most common specialty services were nephrology (2,092, 9.1%), infectious disease (725, 3.1%), gastroenterology (544, 2.4%), and general surgery (371, 1.6%). Among notes where a OL was identified, 5,912 (25.6%) contained documentation of interpreter services use, as defined in the Methods.

### Interpreter service encounter rates

In unadjusted multilevel negative binomial models with LOS included as an exposure (offset) term, inclusion of language preference in the H&P OL was associated with a 15% higher rate of interpreter service encounters per admission day (IRR 1.15, 95% CI 1.02–1.30, *P* = 0.026). In fully adjusted models, this association remained similar (AIRR 1.16, 95% CI 1.03–1.31, *P* = 0.013). When testing for effect modification by language, the interaction between inclusion of language preference and language (Spanish vs. non-Spanish) was not statistically significant (interaction *P* = 0.12), indicating no evidence of heterogeneity in the association by language.

### Documentation of interpreter services

In adjusted multilevel logistic regression models, inclusion of language preference in the H&P OL was associated with higher odds of documented interpreter service use in all notes (AOR 1.71, 95% CI 1.40–2.08, Table 2). Among specialist notes (ie, excluding notes from the general medicine service), the overall adjusted association was not statistically significant (AOR 1.22, 95% CI 0.79–1.90). Effect modification by language category was statistically significant across models (all notes: adjusted model *P* for interaction < 0.001; specialist notes: adjusted model *P* = 0.012). Stratified analysis showed no significant association among Spanish-speaking patients. In contrast, inclusion of language preference was associated with higher odds of documented interpreter services use among non-Spanish-speaking patients (all notes: AOR 2.96, 95% CI 2.18–4.01; specialist notes: AOR 1.74, 95% CI 1.13–2.97).

**Table 2:** Unadjusted and adjusted associations between inclusion of language preference in the H&P one-liners and documentation of interpreter services (Reference: H&P OL does not include language preference). Abbreviation: UOR, unadjusted odds ratio; AOR, adjusted odds ratio

Measure	UOR	95% CI	*P* value	AOR	95% CI	*P* value
**Interpreter Documentation in All Notes**						
Overall	1.88	(1.54, 2.29)	< 0.001	1.71	(1.40, 2.08)	< 0.001
Spanish language preference	1.12	(0.86, 1.46)	0.41	1.04	(0.80, 1.35)	0.78
Non-Spanish language preference	2.87	(2.12, 3.89)	< 0.001	2.96	(2.18, 4.01)	< 0.001
**Interpreter Documentation in Specialist Notes**						
Overall	1.2	(0.78, 1.86)	0.41	1.22	(0.79, 1.90)	0.37
Spanish language preference	0.54	(0.26, 1.14)	0.10	0.70	(0.33, 1.47)	0.38
Non-Spanish language preference	1.66	(0.98, 2.80)	0.06	1.74	(1.13, 2.97)	0.045

## Discussion

In this large retrospective study of inpatients with NELP, the inclusion of language preference in the admitting team’s H&P OL was associated with higher rates of interpreter services use and greater documentation of interpreter encounters during hospital admissions. Given the relatively low overall documentation of interpreter services, this finding points to an opportunity for system-level improvements in how language needs are made visible within routine clinical workflows [19].

The OL is a routinely referenced component of clinical notes and may serve as a non-interruptive cue for language preference embedded within standard workflows. Prior efforts to modify clinician behavior through EHR-based nudges have shown mixed results [20], yet even subtle design features can influence decision making, sometimes subconsciously [21, 22]. For example, Montoy et al. showed that changing the default number of narcotic pills in the EHR directly affected prescribing behavior [23]. In contrast to external alerts or sidebar flags, embedding language preference directly within core clinical documentation may represent a more sustainable and contextually integrated approach to supporting interpreter engagement.

Several mechanisms could explain our results. The H&P written by the admitting team is arguably one of the more accessed notes during an admission, since it generally provides a thorough history of present illness as well as the medical team’s initial reasoning and interventions. Given that the OL in the H&P is highly visible, including a patient’s language preference may heighten clinicians’ awareness of and sensitivity to language needs before meeting the patient, potentially contributing to both higher interpreter encounter rates and more consistent documentation of interpreter services use. The stronger association we observed for documentation of interpreter services among patients with non-Spanish language preference may reflect lower clinician bilingual capacity for these languages (and thus greater reliance on formal interpretation). In contrast, the absence of statistically significant associations for documentation among Spanish-speaking patients may reflect higher rates of clinician Spanish proficiency [24] and less reliance on formal interpreter services in this group.

Importantly, more work is needed to explore if documenting a patient’s preferred language may unintentionally introduce bias by triggering implicit assumptions about their race, ethnicity, or immigrant status. Since language may act as a proxy for race and ethnicity [25], its mention in the OL may lead to similar harmful cognitive biases documented with racial identifiers [26]. Additionally, language-based stigma may arise independently of race, fueled by xenophobic attitudes or stereotypes about non-English speakers, potentially influencing care delivery [15]. Future studies that incorporate patient perspectives are needed to explore these potential harms and any implementation of increasing NELP visibility must center equity, safeguards against stigma, and integrate training around implicit bias.

Limitations include the observational design, which limits causal inference. The use of EHR data introduces potential misclassification due to potential inconsistent documentation of interpreter services. Reliance on billing records may underestimate interpreter services use if services were unbilled or not linked to specific encounters; however, we have no evidence of differential bias, as note authors do not bill for these services. Additionally, preferred language recorded in the EHR may misclassify some patients’ true language preference; however, such misclassification would likely bias results toward the null, as patients would be less likely to require interpreter services. Behavioral confounding is possible, as clinicians who are more attentive to language needs may be both more likely to document NELP in the OL and to use interpreter services. To mitigate this, we included author-level random effects and adjusted for author specialty and service category. Lastly, despite the use of multilevel modeling to adjust for provider-, patient-, and admission-level factors, unmeasured confounding may persist.

This study demonstrates the scalability validated NLP and LLM-based approaches for identifying interpreter documentation in unstructured notes. Compared with structured EHR fields or manual chart review, these methods allow efficient, reproducible analysis at scale and may inform qualify improvement efforts. Future work should evaluate their implementation on equitable language access.

## Conclusion

This study employs novel uses of NLP and LLMs to analyze OLs and interpreter documentation by leveraging a large, real-world dataset that yields robust observational insights. We found the inclusion of a patient’s language preference in the OLs of medical notes to be associated with an increase in interpreter services encounters rates and improved documentation of interpreter services use, particularly among non-Spanish-speaking patients. These findings suggest that incorporating patients’ language preference into core clinical documentation, in addition to existing EHR flags, may represent a scalable, low-burden approach associated with increased interpreter engagement during hospitalization. Prospective evaluation is needed to determine whether this documentation practice meaningfully improves patient-centered outcomes and can be implemented equitably across care settings.

## Data Availability

The datasets generated and/or analyzed during the current study are not publicly available due to institutional data sharing restrictions and patient privacy considerations.

## Abbreviations

NELP: Non-English Language Preference
OL: One-Liner
H&P: History and Physical Note
LLM: Large Language Model
NLP: Natural Language Processing
RPDR: Research Patient Data Registry
EDW: Enterprise Data Warehouse
EHR: Electronic Health Record
HIPAA: Health Insurance Portability and Accountability Act
LOS: Length of Stay
CCI: Charlson Comorbidity Index
